# Skin Substitute Preparation Method Induces Immunomodulatory Changes in Co-Incubated Cells through Collagen Modification

**DOI:** 10.3390/pharmaceutics13122164

**Published:** 2021-12-15

**Authors:** Jordan Holl, Cezary Pawlukianiec, Javier Corton Ruiz, Dawid Groth, Kamil Grubczak, Hady Razak Hady, Jacek Dadan, Joanna Reszec, Slawomir Czaban, Cezary Kowalewski, Marcin Moniuszko, Andrzej Eljaszewicz

**Affiliations:** 1 Department of Regenerative Medicine and Immune Regulation, Faculty of Medicine, Medical University of Bialystok, 15-269 Białystok, Poland; jordan.holl@umb.edu.pl (J.H.); cezary.pawlukianiec@gmail.com (C.P.); javiercortonruiz@gmail.com (J.C.R.); dawid.groth@umb.edu.pl (D.G.); kamil.grubczak@umb.edu.pl (K.G.); 2 1st Clinical Department of General and Endocrine Surgery, Faculty of Medicine, Medical University of Białystok, 15-276 Białystok, Poland; hadyrazakh@wp.pl (H.R.H.); jacek.dadan@umb.edu.pl (J.D.); 3 Department of Medical Pathomorphology, Faculty of Medicine, Medical University of Białystok, 15-269 Białystok, Poland; joasia@umb.edu.pl; 4 Department of Anesthesiology & Intensive Therapy, Faculty of Medicine, Medical University of Białystok, 15-276 Białystok, Poland; slawomir.czaban@umb.edu.pl; 5 Department of Dermatology and Immunodermatology, Faculty of Medicine, Medical University of Warsaw, 02-091 Warsaw, Poland; ckowalewski@wum.edu.pl; 6 Department of Allergology and Internal Medicine, Faculty of Health Sciences, Medical University of Bialystok, 15-276 Bialystok, Poland

**Keywords:** skin substitute, acellular dermal matrix, preparation method, collagen structure, collagen adhesion, dermal architecture

## Abstract

Chronic ulcerative and hard-healing wounds are a growing global concern. Skin substitutes, including acellular dermal matrices (ADMs), have shown beneficial effects in healing processes. Presently, the vast majority of currently available ADMs are processed from xenobiotic or cadaveric skin. Here we propose a novel strategy for ADM preparation from human abdominoplasty-derived skin. Skin was processed using three different methods of decellularization involving the use of ionic detergent (sodium dodecyl sulfate; SDS, in hADM 1), non-ionic detergent (Triton X-100 in hADM 2), and a combination of recombinant trypsin and Triton X-100 (in hADM 3). We next evaluated the immunogenicity and immunomodulatory properties of this novel hADM by using an in vitro model of peripheral blood mononuclear cell culture, flow cytometry, and cytokine assays. We found that similarly sourced but differentially processed hADMs possess distinct immunogenicity. hADM 1 showed no immunogenic effects as evidenced by low T cell proliferation and no significant change in cytokine profile. In contrast, hADMs 2 and 3 showed relatively higher immunogenicity. Moreover, our novel hADMs exerted no effect on T cell composition after three-day of coincubation. However, we observed significant changes in the composition of monocytes, indicating their maturation toward a phenotype possessing anti-inflammatory and pro-angiogenic properties. Taken together, we showed here that abdominoplasty skin is suitable for hADM manufacturing. More importantly, the use of SDS-based protocols for the purposes of dermal matrix decellularization allows for the preparation of non-immunogenic scaffolds with high therapeutic potential. Despite these encouraging results, further studies are needed to evaluate the beneficial effects of our hADM 1 on deep and hard-healing wounds.

## 1. Introduction

Chronic and hard-healing wounds are a pervasive global health concern. The elevated mortality [1] and reduced quality of life associated with these types of wounds, combined with presently limited therapeutic options, highlight the need for novel ameliorative therapies to improve these healing processes.

Wound healing is composed of four overlapping phases: hemostasis, inflammation, proliferation and remodeling [2]. In each of these phases, distinct mechanisms take place to induce effective wound closure and scar formation. During coagulation in the hemostasis phase, the inflammatory cascade is initiated by the degranulation of mast cells, with the central role of wound-infiltrating inflammatory cells such as neutrophils and monocytes clearing the wound of debris and pathogens. Next, the proliferative ability of epidermal keratinocytes and extracellular matrix (ECM)-depositors, including myofibroblasts, facilitates the rapid coverage of the wound area by dermal and epidermal tissue, inducing scar formation. Following wound closure, the remodeling of crucial ECM components takes place, resulting in scar maturation. However, in contrast to normal wound healing, chronic wounds possess a variety of mechanistic dysfunctions, which result in prolonged or ultimately non-existent wound healing [3,4]. To date, several hallmarks of chronic wounds have been identified, such as persistent inflammation [3,4,5,6], low levels of growth factors and high oxidative stress [7,8,9,10], poor angiogenesis [11,12,13], high levels of matrix metalloproteinases [14,15,16,17] and the dysfunction of dermal fibroblasts [10,18]. In fact, proliferative coverage of the wound area is significantly inhibited in chronic wounds, and the healing process is blocked in a self-perpetuating cycle of inflammatory reaction. Therefore, it is well recognized that the ideal dressing for hard-healing wounds should not only cover the wound but also modulate the wound microenvironment through its immunoregulatory effects.

Notably, skin substitutes or skin-derived dressings have been used as an effective complementary treatment for deep, extensive, and hard-healing wounds, among others [19,20,21,22,23,24,25,26,27]. Skin substitutes are biologically or synthetically derived tissues applied to the wound to induce favorable wound healing effects. In fact, skin substitutes have a long history of use in treating wounds and ulcers of different etiology. However, their therapeutic effects depend on the derivative source, structural composition, preparation method, and means of sterilization/aseptic creation, among others [2,26,28].

Recent studies demonstrated the regenerative potential of ADM application in diabetic foot ulcer (DFU) healing and mechanisms associated with beneficial effects [22,29,30,31]. Moreover, the members of our group have developed a novel approach to hADM manufacturing from the resected skin fold of living abdominoplastic surgery patients. Previously, we were able to generate a novel abdominoplasty skin-derived hADM using a distinct method of decellularization and sterilization. We previously evaluated the effects of different methods of preparation on the purity and structure of our novel hADM. Moreover, we showed that human abdominoplasty skin-derived ADMs may serve as dressing for deep wound treatment [32]. More importantly, this dermal matrix was able to serve as a skin substitute for hard-healing wounds or as a biological scaffold for BIOOPA dressing to treat epidermolysis bullosa patients [23,28,33,34]. Here, we examined three differentially prepared hADMs and aimed to evaluate how these methods of aseptic preparation may influence their immunomodulatory properties and therapeutic potential.

## 2. Materials and Methods

### 2.1. Skin Collection and Processing

Skin folds from bariatric patients were collected during abdominoplastic surgery. Dermatome skin grafts were harvested from the resected skin fold, sealed in foil bags, and biobanked at −80 °C for further processing.

Dermal fragments were thawed in saline at room temperature, followed by a decellularization step using chemical and/or enzymatic processing (Supplementary Table S1). Directly after decellularization and washing, acellular dermal matrices were lyophilized using automated −80 °C lyophilization, sealed in double foil bags, labeled, and biobanked in −80 °C. The efficiency of the decellularization process was controlled using histochemical staining.

Prior to use, 8 mm fragments were created by biopsy punch. Next, 8 mm acellular dermal matrix (ADM) fragments were re-hydrated in a 1 mL complete culture medium RPMI 1640 (Thermo Fisher, Waltham, MA, USA) supplemented with 10% FBS (PAN Biotech, Aidenbach, Germany) and 75 μg/mL gentamicin (Gibco, Waltham, MA, USA) for 24 h in a 37 °C, 5% CO_2_ incubator. Each independent experiment used an hADM from a distinct decellularization series.

### 2.2. Peripheral Blood Mononuclear Cell (PBMC) Isolation

PBMCs were isolated from fresh buffy coats obtained from healthy donors by means of density gradient centrifugation (Pancol, PAN Biotech), as previously described [28]. Pharm lyse buffer (BD, Franklin Lakes, NJ, USA) was used to remove residual red blood cells when needed. PBMC numbers were evaluated using a Bürker chamber. Freshly isolated PBMCs were resuspended in complete culture medium and used immediately for further research. Buffy coats were collected upon the approval of the Ethics Committee of the Medical University of Bialystok.

### 2.3. T Cell Proliferation Assay

Freshly isolated PBMCs were stained with carboxyfluorescein succinimidyl ester (CFSE, Sigma-Aldrich, St. Louis, MO, USA) in PBS (Corning Inc., Corning, NY, USA) for 5 min at room temperature in the dark. CFSE-labeled cells were washed three times in PBS (5 min, 400× *g*). Next, the cells were resuspended in complete culture medium and gently seeded on re-hydrated 8 mm hADM fragments, 8 mm unprocessed skin fragments, or left alone (vehicle/unstimulated control) in 24 well culture plates (Eppendorf, Hamburg, Germany) at the density of 1 × 106 cells/m. Mitogen stimulation (5 μg/mL PHA-P, Gibco) was included as a positive control of PBMC proliferation. The cells were stimulated for 7 or 14 days with medium changes every four days. Finally, the cells were collected and stained with mouse anti-human CD3-FITC and CD8-PE conjugated antibodies (BD Biosciences) for 15 min in the dark. See Supplementary Table S2 for antibody information. Next, the samples were washed in PBS and fixed using CellFix (BD) and analyzed by FACSCalibur (BD) flow cytometry. Obtained data were analyzed using FlowJo v10 software (TreeStar Inc., Ashland, OR, USA). Appropriate staining controls were used for setting the gates. The used gating strategy is presented in Supplementary Figure S1. The results are presented as a ratio of proliferation versus the unstimulated control.

### 2.4. Assessment of Monocyte and T Cell Phenotype

Freshly isolated PBMCs were gently seeded on re-hydrated 8 mm hADM fragments or left alone (vehicle/unstimulated control) in 24 well culture plates (Eppendorf) at the density of 1 × 106 cells/mL in complete cell culture media. The cells were cultured for up to 72 h and collected every 24 h for flow cytometry analysis. For intracellular cytokine staining, brefeldin A (Thermo Fisher) was added to culture wells 3 h before cell acquisition. Additionally, cell culture supernatant was collected and biobanked at −80 °C for cytokine assay. Next, the cells were stained immediately with a panel of monoclonal antibodies (Supplementary Table S2). Briefly, cells were incubated in the presence of monoclonal antibodies for 30 min at room temperature in the dark. Next, the specimens were washed twice in PBS and fixed with CellFix (BD) or subjected to intracellular staining. For the latter, samples were fixed and permeabilized using Perm2 Buffer (BD) according to manufacturer instructions, followed by washing in PBS and staining with fluorescent-conjugated antibodies (Supplementary Table S2) for 30 min at 4 °C in the dark. Next, the cells were washed twice and fixed with CellFix (BD). Finally, the specimens were analyzed on a FACSCalibur flow cytometer (BD). Flow cytometry data analysis was performed using FlowJo software (Tree Star). Appropriate, fluorescence-minus-one (FMO) controls were applied for setting correct gating. Used gating strategies for different T cell and monocyte subsets are presented in Supplementary Figures S2 and S3. Used gating strategies for different T cell and monocyte functions are presented in Supplementary Figures S4 and S5.

### 2.5. Cytokine Assay

Concentrations of factors TNF, IFN**γ**, IL-1β, IL-6, IL-10, IL-17 and TGF-β were measured by means of commercially available DuoSet ELISA (all from R&D Systems, Minneapolis, MN, USA), as previously described [28,34,35]. The detection ranges for TNF (15.6–1000 pg/mL) IFN**γ** (9.39–600 pg/mL), IL-1β (3.91–125 pg/mL), IL-6 (9.4–600 pg/mL), IL-10 (31.3–2000 pg/mL), IL-17 (15.6–1000 pg/mL) and TGF-β (31.3–2000 pg/mL). Protein levels were analyzed with an automated microplate reader (LEDETEC96 system). The results were calculated according to the standard curve, generated by individual standard dilutions, by MicroWin 2000 Software.

### 2.6. Immunofluorescence Staining

hADMs were snap-frozen after coincubation with PBMCs mentioned previously. hADMs were cut using cryomicrotome into 20 μM longitudinal sections and seeded on glass slides. Next, cryosections were fixed with 4% paraformaldehyde (Santa Cruz Biotechnology, Dallas, TX, USA) and incubated in a detergent (0.1% Triton X-100 (Sigma)) in 0.02% SDS-PBS (Sigma-Aldrich and Corning, respectively), followed by incubation with blocking buffer (10% normal donkey serum–Abcam, Cambridge, UK) in 1% BSA in PBS). Next, the slides were stained with a specific primary antibody for collagen types I, III and IV or incubated in staining buffer (1% protease-free bovine serum albumin (Sigma) in PBS) for 60 min in a high humidity chamber in the dark. Next, the slides were washed three times in washing buffer (Tween20-PBS) and stained with appropriate secondary antibodies. For detailed characteristics of primary and related secondary antibodies, please see Supplementary Table S3. Finally, the specimens were mounted in Prolong Gold mounting medium with DAPI (Thermo Fisher) and covered with cover slides (Avantor, Gliwice, Poland), followed by overnight incubation at RT in the dark before analysis by confocal microscopy.

### 2.7. Confocal Microscopy

Confocal pictures were acquired using an FV1200 Microscope (Olympus, Tokyo, Japan). Full-sized pictures were acquired in 3 channels using 405, 450 and 650 nm lasers at 20 μs/pixel and 2048 × 2048 size using FluoView software (https://www.olympus-lifescience.com; Olympus) accessed on 1 March 2021. Full-sized photos were then examined using ImageJ software (https://imagej.nih.gov/ij/; Public domain) accessed on 1 May 2021. Channel 1 (DAPI) was made blue, Channel 2 (autofluorescent collagen structure) was made green, Channel 3 (stained collagen fibers I, III or IV) was made red. Z-stacks of each channel were created and merged. As many focused sections (500 × 500 pixels) as possible were extracted from each full-sized photograph inside the hADM for collagens I and III or on the apical edge for collagen IV, and quantified. Each stained, quantified, focused section was divided by the mean of combined focused controls; slides stained without primary collagen-binding antibody but with secondary fluorescent antibodies specific to each collagen [Quantification = Stained Collagen/Staining Control]. Channels 1 and 2 (DAPI and autofluorescence, respectively) did not contribute to the semi-quantitative measurement of collagens. In hADMs without co-incubated PBMCs, only one time point (24 h) was used with quantification measured as mentioned above. However, when PBMC co-incubated hADMs were compared to those without PBMCs, the following equation was used [Quantification = (stained PBMC co-incubated focus section/control PBMC co-incubated focus section)/(stained non-PBMC focus section/control non-PBMC focus section)].

### 2.8. Quantification of DNA Present within hADMs

hADMs were re-hydrated using 1 mL of PBS in 24 well plates. Next, hADMs were digested using proteinase K in buffer solution provided in DNeasy blood and tissue DNA isolation kit (Qiagen, Hilden, Germany). This resulted in the entire dissolution of hADMs into solution. This kit was used according to manufacturer protocol to isolate and extract DNA from the fibrous hADMs. Finally, DNA concentration was measured using a NanoDrop™ 2000/2000c Spectrophotometer (Thermo Fisher) using NanoDrop 2000 software (http://isogen.nl/nanodrop-software; Thermo Fisher) accessed on 3 October 2021. Specifically, the nucleic acid measurement at 260/280 nm was used.

### 2.9. Statistics

Graphs and statistics were calculated using GraphPad Prism 8 (http://graphpad.com; GraphPad Software, San Diego, CA, USA) accessed on 1 August 2020. Wilcoxon matched-pairs signed-rank test was used to compare differences between analyzed conditions. To determinate the differences in residual DNA, Mann-Whitney U-test was used. The differences were considered statistically significant at *p* < 0.05. The results are presented as a median ± interquartile range.

## 3. Results

### 3.1. Different Methods of Human Abdominoplasty Skin Preparation Influence Acellular Dermal Matrix Immunogenicity

It is implied that an ideal skin substitute or dermal dressing should be low- or non-immunogenic and support healing processes [2]. However, due to the nature of tissue sourcing, method of processing, and the sterilization that ADMs undergo, various allogenic tissue-derived immunogenic mediators may be retained within and later released from their structure after wound implantation. Therefore, we first aimed to analyze whether aseptic preparation procedures of our novel abdominoplasty skin-derived ADMs may affect their immunogenicity.

**Figure 1:**
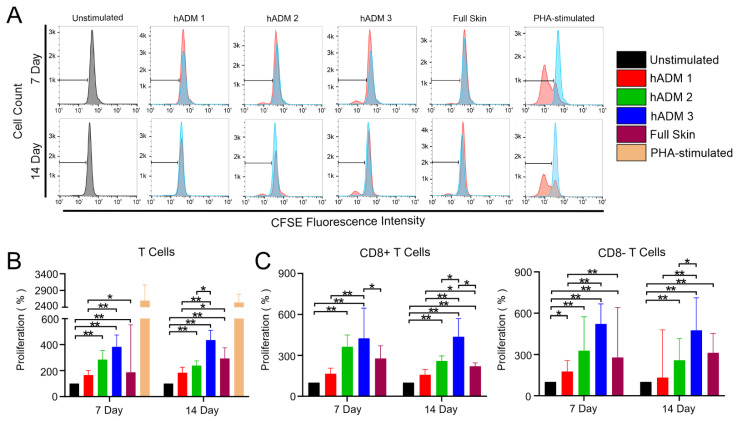
hADM preparation method induces differential T cell proliferation. Flow cytometric analysis of T cell proliferation. CFSE-stained PBMCs were co-incubated alone, with hADMs, with full skin, or with mitogenic control (lectin PHA) for 7 or 14 days. (**A**) Histograms displaying proliferation of whole T cells for 7 and 14 days. (**B**) Quantification of whole CD3+ T cell proliferation. (**C**) Quantification of CD3+/CD8+ and CD3+/CD8− T cell proliferation. Results expressed as medians + interquartile ranges. Two-tailed Wilcoxon matched-pairs signed-rank test used for (**B**,**C**). *n* = 5 with 2 technical replicates; * *p* < 0.05. ** *p* < 0.01.

First, we used a CFSE-based assay to analyze the proliferation of CD3+ T cells and their two main subsets, namely cytotoxic (CD3+CD8+ T cells) and helper (CD3+CD8−) T cells (Figure 1A and Supplementary Figure S2). hADM 1 had low immunogenicity and did not induce T cell proliferation, as no significant differences were observed when compared to the unstimulated control in both analyzed time points (Figure 1B). In contrast, hADM 2 and hADM 3 induced greater T cell proliferation comparable to unprocessed skin. Moreover, hADM 3 showed the highest immunogenicity among all tested groups (Figure 1B). Moreover, the same differences were observed in both cytotoxic and helper T cells (Figure 1C).

Taken together, we showed here that different method of novel human abdominoplasty skin-derived ADM preparation can influence their immunogenicity and induce T cell proliferation. Further, hADM 1 induced the least immunogenic effect in co-incubated T cells, indicating putatively beneficial effects.

### 3.2. Abdominoplasty Skin-Derived ADM Preparation Methods Do Not Influence T Cell Phenotype

Recent evidence indicates the crucial role of T cells in wound healing and scar formation, showing that T cell depletion significantly impairs the healing process and increases scar size [36,37,38]. Therefore, having found that the differential preparation method of hADMs affects immunogenic T cell proliferation, we next wished to analyze whether observed differential T cell responses are associated with changes in their phenotype in the early stage of coincubation mimicking the inflammatory phase of wound healing.

First, we aimed to investigate changes in the composition of different T cell subsets, namely non-activated T cells (CD4+/CD25−/CD127+), activated T cells (CD4+/CD25+CD127+), putative regulatory T cells (CD4+/CD25+/CD127low/−), and putative Th17 cells (CD4+/CD161+/CD196+) (Supplementary Figure S2A). We found no significant differences in the frequency of non-activated, activated, and putative Th17 cells (Supplementary Figure S2B). However, the frequency of putative Tregs was slightly increased at 24 and 48 h, but not 72 h (Supplementary Figure S2B). Next, we aimed to analyze whether the manufacturing procedure of abdominoplasty skin-derived hADM may influence the frequency of pro-inflammatory IFN**γ** and IL-17 producing T cells incubated in the presence of our novel hADMs. However, we observed no significant differences in the frequency of analyzed subsets (Supplementary Figure S4B). Similarly, no differences were observed in the mean fluorescence intensity of IFN**γ** and IL-17 in T cells (Supplementary Figure S4C).

Taken together, we showed that the differential abdominoplasty skin-derived hADM preparation method does not significantly influence T cell phenotype composition.

### 3.3. Novel Abdominoplasty-Derived Skin Substitutes Cause Differential Monocyte Activation

Given monocytes/macrophages central role in all phases of wound healing and their ability to orchestrate adaptive immune responses in the process of antigen presentation (including T cell proliferation induction) [39,40,41,42,43], we wished to evaluate the activation of monocytes after coincubation with differentially manufactured abdominoplasty skin-derived hADMs, hypothesizing that they may influence the previously witnessed differential T cell proliferation.

**Figure 2:**
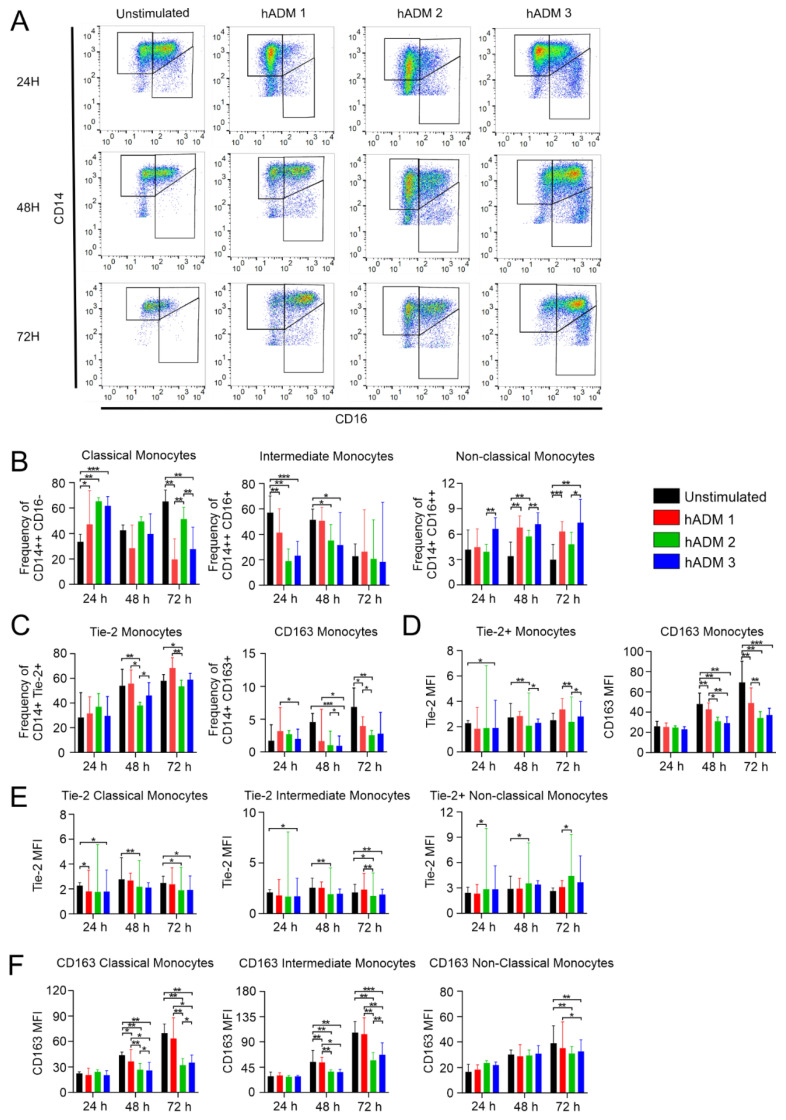
Monocyte phenotype is altered by hADM preparation method. Healthy donor PBMCs were co-incubated alone or with hADMs for 1, 2 or 3 days. Next, cells were stained extracellularly and examined by flow cytometry. (**A**) Representative CD14 × CD16 gating plots for each unstimulated and hADM condition at 24 h, 48 h and 72 h. (**B**) Subset frequencies for classical (CD14++/CD16−), intermediate (CD14++/CD16+), and non-classical (CD14+/CD16++) monocytes. (**C**) Frequency of CD14+/Tie-2 or CD163+ monocytes. (**D**) MFI quantification of CD14+ monocytes for Tie-2 or CD163. (**E**) Tie-2 MFI for specific monocyte subsets. (**F**) CD163 MFI for specific monocyte subsets. Results expressed as medians + interquartile ranges. Two-tailed Wilcoxon matched-pairs signed-rank test used for (**B**–**F**) *n* = 5 with 2 technical replicates; * *p* < 0.05, ** *p* < 0.01, *** *p* < 0.001.

First, we aimed to analyze the composition of three major monocyte subsets, namely the frequency of classical (CD14++CD16−, non-activated), intermediate (CD14++CD16+), and non-classical (CD14+CD16++) monocytes (Figure 2A). In fact, we found significant differences in the composition of monocyte subsets after incubation with all analyzed hADMs compared to the unstimulated control (Figure 2B). The frequency of classical monocytes was higher in all analyzed conditions at 24 h. Consequently, we found a lower frequency of activated monocytes (cells with CD16 expression), namely intermediate but not non-classical monocytes (Figure 2B). Contrastingly, no significant differences in the frequency of classical monocytes were observed at 48 h; however, we found a significantly lower frequency of intermediate monocytes after incubation with hADM 2 and hADM 3. Moreover, a higher frequency of non-classical cells was observed after incubation with hADM 1 and hADM 2, but not hADM 3 (Figure 2B). At 72 h, we observed a significantly lower frequency of classical monocytes after incubation with hADM 1 and hADM 3. No differences were observed in intermediate monocytes, while non-classical monocyte frequency was higher in all analyzed conditions when compared to the unstimulated control (Figure 2B).

Having found significant changes in the composition of different monocyte subsets, we next sought to analyze whether these observed differences were associated with the frequency of Tie-2- and/or CD163-expressing cells, these markers being associated with pro-angiogenic and anti-inflammatory properties, respectively [44,45]. In addition, we wished to analyze the frequency of anti-inflammatory IL-10 and pro-inflammatory TNF-producing monocytes.

**Figure 3:**
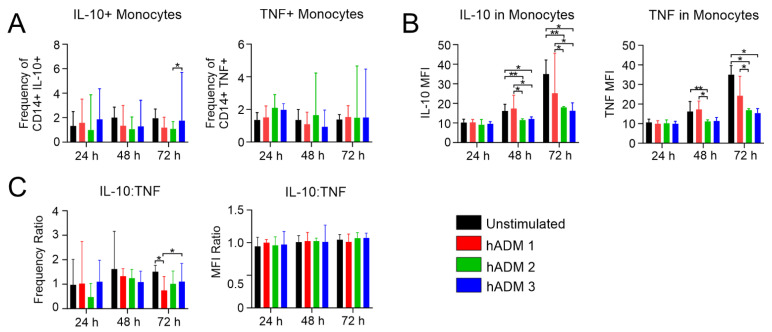
Monocyte effector function is modulated by hADM coincubation. Healthy donor PBMCs were co-incubated alone or with hADMs for 1, 2, or 3 days. A total of 2 h before collection, protein transportation was inhibited. Finally, cells were permeabilized and stained extracellularly and intracellularly before being examined by flow cytometry. (**A**) Frequency of CD14+/IL-10+ or TNF+ monocytes. (**B**) MFI quantification of CD14+/IL-10+ or TNF+ monocytes. (**C**) Frequency and MFI ratios of IL-10:TNF. Results expressed as medians + interquartile ranges. Two-tailed Wilcoxon matched-pairs signed-rank test used for (**A**–**C**) *n* = 5 with 2 technical replicates; * *p* = 0.05, ** *p* = 0.01.

We found no differences in the frequency of Tie-2-expressing monocytes after 24 h incubation (Figure 2C). However, at 48 h, the frequency of analyzed cells was lower after incubation with hADM 2, while no differences were observed at 72 h compared to the unstimulated cells (Figure 2C). The frequency of CD163-expressing monocytes did not change after 24 h stimulation when compared to the unstimulated control. However, at 48 h, we found a lower frequency of analyzed cells incubated with hADM 3 compared to the unstimulated control, and similarly at 72 h in cells incubated with hADM 2 and hADM 3 (Figure 2C). Similarly, no differences in CD163 expression were observed at 24 h. In the latter time points, we found a significantly lower expression level of CD163 on monocytes when compared to unstimulated control. Interestingly, cells incubated with hADM 2 and hADM 3 showed lower levels of analyzed molecules when compared to hADM 1 (Figure 2D). Unexpectedly we found no differences in the frequency of IL-10 and TNF-producing monocytes (Figure 3B). However, at the 48 and 72 h time points, IL-10 and TNF expression (defined as MFI) was lower in hADM 2 and hADM 3, but not hADM 1 co-incubated cells versus unstimulated control (Figure 3C).

Here we showed how differentially produced hADMs may modulate monocyte maturation, initially delaying the surface expression of CD16 and maturation into intermediate and non-classical subsets when compared to the control. Further, we observed a declining expression of CD163 in hADM co-incubated groups over time and no difference in IL-10 and TNF-producing monocytes. Crucially, the method of hADM preparation can be seen to induce differential monocyte subset polarization.

### 3.4. Differentially Manufactured Abdominoplasty Skin-Derived Acellular Dermal Matrices Induce a Distinct Pattern of Cytokine Responses

**Figure 4:**
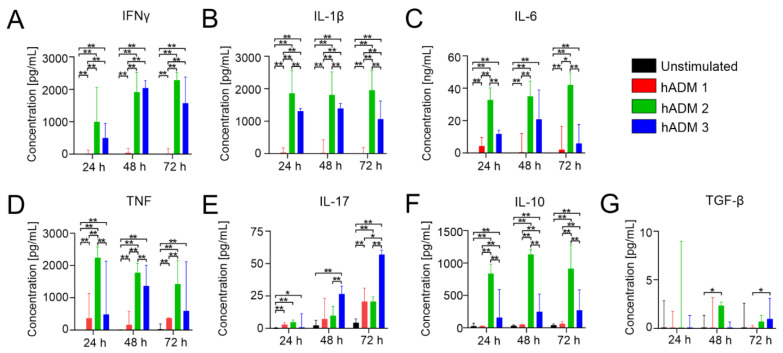
Method of hADM preparation induces differential cytokine secretion in vitro. Healthy donor PBMCs were co-incubated alone or with hADMs for 1, 2, or 3 days. Supernatants were collected, and ELISA was performed on 100 uL fractions. (**A**–**E**) Pro-inflammatory and (**F**,**G**) Anti-inflammatory cytokine and growth factor concentrations. Results expressed as medians + interquartile range. Two-tailed Wilcoxon matched-pairs signed-rank test used for (**A**–**G**) *n* = 6 with 2 technical replicates. * *p* = 0.05, ** *p* = 0.01.

Having found significant changes in T cell proliferation and monocyte activation, we next wished to analyze levels of pro-inflammatory IFN**γ**, IL-1β, IL-6, TNF, IL-17 and anti-inflammatory IL-10 and TGF-β in cell culture supernatants (Figure 4). We found that hADM 2 and hADM 3 induced pro-inflammatory profiles, with high levels of IFNγ, TNF, IL-1β, and IL-6 when compared to both unstimulated control and hADM 1 (Figure 4A–D) in all analyzed time points. In contrast, levels of IL-17 were relatively low at 24 h, with increasing concentrations observed in the later time points. In fact, hADM 3 showed the highest level of IL-17 in analyzed samples. In hADM 2 and hADM 3, we observed a trend to increase IL-17 levels at 48 h, and which reached statistical significance at 72 h time point (Figure 4E). The highest levels of IL-10 were observed in PBMCs after incubation with hADM 2 (Figure 4F). Although hADM 3 induced moderate IL-10 secretion, this was still significantly greater than hADM 1 and the unstimulated control. Finally, TGF-β was measured, but the concentrations observed in the supernatant were very low (Figure 4G). Significantly higher levels of TGF-β were observed only in cell culture supernatants from PBMC incubation with hADM 2 at 48 h and hADM 3 at 72 h time points (Figure 4G).

Ultimately, our results demonstrate the broad pro-inflammatory profiles associated with PBMCs co-incubated with hADMs 2 and 3 in regard to IFN**γ**, IL-1β, IL-6, TNF and IL-17. Contrasting, hADM 1 possessed an attenuated inflammatory cytokine profile. Finally, anti-inflammatory IL-10 levels were high for hADM 2 and moderate for hADM 3, while low in hADM 1 and the control.

### 3.5. hADM Apical Architecture Is Differentially Extracted by Prepatory Method

**Figure 5:**
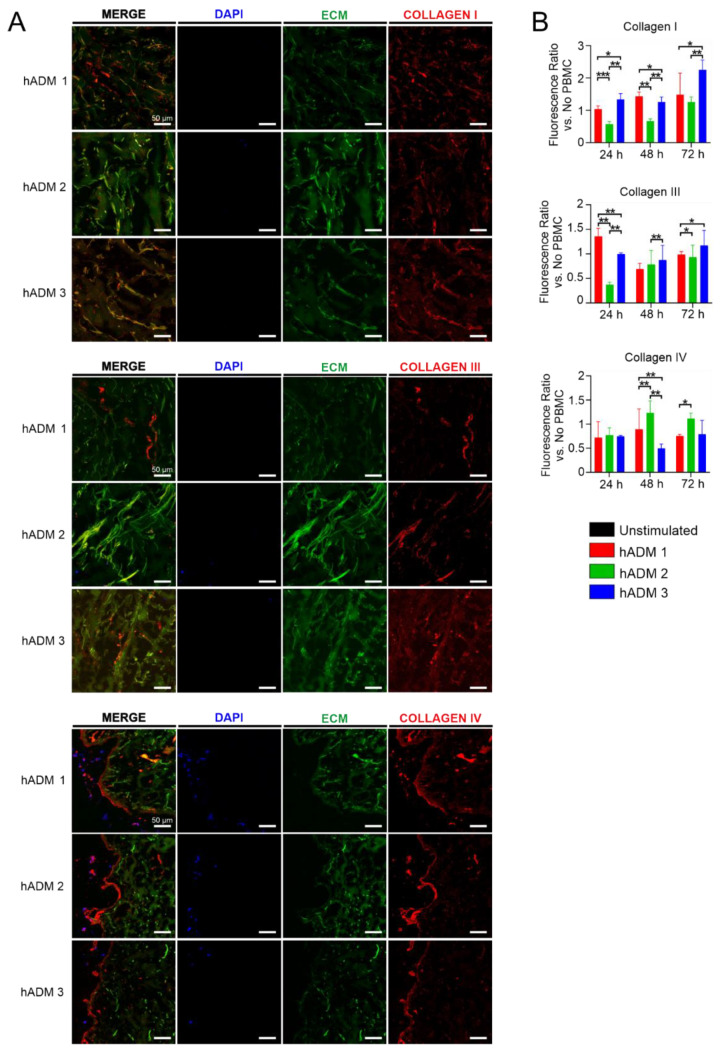
Collagen fibers are retained after coincubation with PBMCs. hADMs were examined confocally after fluorescence-conjugated antibody staining for collagens I, III and IV. Quantifications are derived from focused z-stack photographs using whole channel fluorescence. (**A**) Images for collagens I, III and IV can be seen for each hADM and in 3 separate channels as well as merged together. MERGE: combined representation of channels 1–3, detailed below. Channel 1 (DAPI): cell nuclei staining via DAPI using a 405 nm laser. Channel 2 (ECM): autofluorescent collagen fibers and ECM using a 488 nm laser. Channel 3 (specific collagen): specific collagen fibers were stained using a primary and subsequently a secondary fluorescence-conjugated antibody using a 647 nm laser. Process of acquisition and analysis described fully in Materials and Methods section. (**B**) Comparison of collagens in PBMC co-incubated hADMs vs. hADMs without cell culture. Z-stack sections had their absolute fluorescences divided by control sections. Quantification formula can be seen in the Materials and Methods section. Results expressed as medians + interquartile ranges. Two-tailed Wilcoxon matched-pairs signed-rank test used for B. *n* = 3 with 2 technical replicates; * *p* < 0.05, ** *p* < 0.01, *** *p* < 0.001. All scale bars represent 50 µm.

The dermal layers of the skin are mainly composed of different types of collagen fibers [46,47]. Furthermore, it is well established that immune cells may change collagen architecture by the release of proteolytic enzymes, such as metalloproteinases [17,48,49]. Therefore, having found significant differences in the immune responses to differentially manufactured novel abdominoplasty skin-derived hADMs, we next wished to analyze the influence of immune cells on the extracellular matrix structure, namely collagen architecture (Figure 5).

First, we found no visible differences in the structures of all three analyzed hADMs after coincubation with PBMCs (Figure 5A). PBMCs were located on the apical portion of hADMs and did not penetrate to the deeper layers of the matrix. Interestingly, in the apical site, fragmentation of collagen IV was observed. Moreover, we found the co-localization of mononuclear cells with collagen IV fragments, suggesting phagocytosis of released fibers (Figure 5A). Next, we quantified type I, III and IV collagens after hADM coincubation with PBMCs relative to hADMs incubated alone (Supplementary Figure S6). Interestingly, we observed slight but significant differences in the density of various collagen fibers in all three analyzed time points, namely at 24, 48 and 72 h. The presence of collagen I was lowest in hADM 2 compared to analyzed counterparts in all analyzed time points, while hADM 3 presented the highest content of collagen I after 24 and 72 h incubation. In contrast, collagen III was higher in hADM 1 at 24 h and further decreased at 48 and 72 h when compared to hADM 3. Again, hADM 2 showed the lowest density of collagen III fibers after incubation in the presence of PBMC among analyzed matrices. In contrast, no differences were observed in the density of collagen IV among analyzed hADMs at 24 h, while at 48 h, hADM 2 showed a slightly higher level of this collagen. However, at 72 h, the collagen IV level in hADM 1 was lower when compared to hADM 2.

Here we showed differences in collagen retention in PBMC co-incubated hADMs, finding that collagen I was extracted in hADM 2 at all time points. Further, we found differences in the extraction of collagen III, noting slight extraction of hADM 1 over time. hADM appeared to possess slight collagen III depositions, but this still ultimately left it with the lowest collagen III concentration at 72 h. Finally, we examined collagen IV, finding only small differences in its overall quantification, although significant fragmentation was witnessed.

## 4. Discussion

Here we showed that similarly sourced but differentially manufactured novel human abdominoplasty skin-derived ADMs possess different immunogenic and immunomodulatory properties. Furthermore, our results indicate the potentially favorable immunomodulatory properties of hADM 1, while hADM 2 and hADM 3 seem to be more immunogenic, notably characterized by high inflammatory cytokine profiles.

The preparation of biological scaffolds, including ADMs, aims to remove all cellular and nuclear components from tissue while preserving the three-dimensional ultrastructure of the ECM. In fact, decellularization procedures should be balanced to preserve the delicate structure of the scaffold and remove all unwanted components that may trigger strong inflammatory reactions [50,51]. Notably, these residual components, including nucleic acids, may act as danger-associated molecular patterns that stimulate pattern recognition receptors of the innate immune system, such as toll-like receptors (TLR9), RIG-I-like receptors and AIM2-like receptors [52,53]. A greater quantity of matrix-bound DNA (data presented in Supplementary Figure S7) partially explain the observed increased inflammatory responses of hADMs 2 and 3 co-incubated PBMCs, including the proliferative response of T cells monocyte activation and higher cytokine release. Moreover, it may also explain the unexpectedly observed increase in the proliferation of hADM 2 and 3 co-incubated T cells when compared to unprocessed skin [54].

Nearly 80% of the ECM is composed of insoluble or hardly soluble proteins, mainly collagen, which, upon application to the wound, serves as a scaffold for different cell subsets [48,55,56,57] and improves healing potential [58]. Recent evidence shows that particular compositional elements of the ECM can elicit immune cell activation, which in turn triggers specific cytokine responses in vitro and in vivo [59,60,61]. These immune-modulatory properties may be induced by direct antigenic effects of ECM structure or released components, including collagen fragments, laminin, hyaluronan, and integrins [60,62]. Notably, the use of enzymes with proteolytic activity, such as trypsin and its modifications (as in hADM 3), allows for the cleavage of proteins adherent to cells, thereby separating cellular contents from the ECM. It has been shown that enzymatic decellularization is more destructive to elastin and collagen fibers in comparison to ionic and non-ionic detergents, such as those used in our study (sodium dodecyl sulfate (SDS) and Triton X-100, respectively) [63,64,65,66,67]. This ECM-extracting effect explains the observed higher immunogenicity of hADM 3 in our study. On the other hand, however, the detergents modulate fibrillar collagen structure, which may result in decreased mechanical strength of the end product [68,69,70]. SDS is an anionic detergent that effectively extracts and denatures proteins, allowing for the efficient removal of cellular and antigenic contents of processed tissue [71,72,73]. In addition, SDS has been shown to reduce soluble collagen content by alteration of its molecular structure, bringing it to the point of insolubility [74]. In contrast, Triton X-100 disrupts DNA-protein, lipid-protein, and lipid-lipid interactions while maintaining the native structure of ECM [75,76]. It is believed that the use of both ionic and non-ionic detergents decreases the immunogenicity of decellularized grafts [63,77]. Similarly, in our study, the use of detergents allows for the manufacture of hADMs with relatively low immunogenicity. However, the observed use of SDS seems to be more effective in the reduction in T cell proliferation and cytokine release.

T cell responses are regulated by changes in the composition and function of T cell subsets. They may be regulated by the induction of cellular plasticity (involving epigenetic machinery in response to microenvironmental changes) or induced in the process of antigen presentation by antigen-presenting cells (such as monocytes/macrophages and dendritic cells) [40,41,78,79]. In our study, we found no direct effect of novel human abdominoplasty skin-derived ADMs on differential T cell subset composition. Therefore, we assumed that our hADMs do not directly induce T cell plasticity.

Decellularization procedures can preserve ECM fragments that can regulate cell migration and modulate local inflammatory responses, improving graft integration with recipient tissues and supporting healing mechanisms. In the wound bed, infiltrating monocytes and dermal macrophages play a central role, orchestrating all steps of healing by (a) clearing the wound from pathogens and debris; (b) releasing growth factors-guiding local progenitor cells proliferation and differentiation; (c) producing inflammatory mediators-regulating immune responses and inducing migration of immune cells and progenitor cells to the wound; (d) releasing pro-angiogenic factors-ultimately regulating, both directly and indirectly, tissue neovascularization; and (e) releasing ECM modulating enzymes-contributing to scar maturation [45,79,80,81]. In addition, monocytes/macrophages have been shown to therapeutically modulate pathological processes in hard-healing wounds and ulcers (such as diabetic foot ulcers) treated with ADMs [31,82,83]. Therefore, the ideal dermal dressing should target the pro-inflammatory function of monocytes and macrophages and induce their reparatory properties, among others [84,85].

Peripheral blood monocytes constitute of three functionally distinct cell subsets, namely (a) classical CD14++CD16− monocytes (non-activated cells with high phagocytic activities that, upon activation by cytokines, chemokines, growth factors, the danger-associated or pathogen-associated molecular patterns (DAMPS and PAMPS, respectively) acquire CD16 expression); (b) intermediate (CD14++CD16+) monocytes (putative precursors of alternatively activated macrophages referred to as M2 cells with high reparatory and anti-inflammatory potential); and (c) non-classical (CD14+CD16++) monocytes (putative precursors of classically activated M1 macrophages with high pro-inflammatory potential) [86,87]. Notably, in vitro monocyte maturation toward macrophages can be induced by their adhesion to plastic or glass surfaces [88]. Similarly, in our study, monocytes incubated without the presence of hADMs start their maturation process, which was observed as a significant decrease in classical monocyte frequency and the subsequent increase in CD16+ cells. It seems, however, that initially (at 24 h), our novel hADMs reduce the activation and maturation of monocytes toward macrophage-like cells. However, in the later time points, maturation was induced predominantly toward cells with reparatory and pro-angiogenic potential, namely intermediate monocytes and Tie-2 expressing cells [45,89]. It is well established that the implementation of M2 macrophages to biomaterials improves vascularization and healing more effectively when compared to M1-like cells [90,91,92]. However, to date, the mechanistic role of ECM components in monocyte differentiation toward macrophages and their polarization remains elusive. Recent evidence shows that decellularized ECM supports the generation of M2 monocyte-derived macrophages with different CD23, CD163 and EGR2 expressions [31]. It seems that monocytes differentiate on the surface of ECM and do not penetrate to its deeper layers [93]. Although this is also broadly supported by our observations, in some contrast to previous reports, we found no increase in the frequency of CD163-expressing monocytes. This may be associated with a relatively short culture period and/or a different source of decellularized tissue. Moreover, we found that the hADM surface is partially degraded by collagen IV release, which is further digested by monocytes. In some contrast, collagen I and III seem to remain intact.

Finally, the distinct cytokine profiles induced by differential hADM production must be acknowledged. Previously, an in vitro model of sterile tissue inflammation that induced pro-inflammatory M1-like effects in macrophages was able to be abrogated by coincubation with an artificial dermal matrix composed of collagen and modified hyaluronan [94], in addition to the aforementioned immunomodulatory effects witnessed in macrophage interactions with integrins [61]. Therefore, the observed elevated inflammatory cytokine expression in hADM 2 and 3 co-incubated PBMCs appears consistent with M1-like cell activation, providing a compelling future target in the deeper examination of modified and/or extracted ECM components that may induce distinct immunomodulatory effects. Taken together, we showed here that decellularization of human abdominoplasty skin using anionic detergent (SDS) could be used for novel non-immunogenic hADM preparation (hADM 1). Given its high potential to induce monocyte polarization toward anti-inflammatory/reparatory M2-like cells, hADM 1 represents a candidate who can serve as a dressing for deep, hard-healing, and/or chronic wounds. However, further in vivo studies are needed to elucidate its therapeutic potential. Moreover, there is still a substantial need to better understand the effects of different decellularization procedures and the role of constituent structural components of decellularized ECM regarding their ability to improve wound healing processes.

## Data Availability

All data and research materials are available at reasonable request from the corresponding authors.

## Supplementary Materials

The following are available online at https://www.mdpi.com/article/10.3390/pharmaceutics13122164/s1, Figure S1: Representative gating for T cell proliferation, Figure S2: T cell phenotype is not regulated by hADM coincubation, Figure S3: Representative gating for extracellularly stained monocytes, Figure S4: hADMs do not induce differential T cell function, Figure S5: Representative gating strategy for intracellularly stained monocytes, Figure S6: Representative photos of ADMs without PBMC coincubation, Figure S7: Quantification of residual DNA, Table S1: hADM decellularization protocols, Table S2: Antibodies used in flow cytometry, Table S3: Antibodies used in confocal microscopy.

## Abbreviations

CFSE	Carboxyfluorescein Succinimidyl Ester
ECM	Extracellular Matrix
hADM	Human-derived Acellular Dermal Matrix
IFNγ	Interferon Gamma
IL	Interleukin
LPS	Lipopolysaccharide
MFI	Mean Fluorescence Intensity
PBMC	Peripheral Blood Mononuclear Cells
TGF-β	Transforming Growth Factor Beta
Th1/17	T helper Types 1 or 17, respectively
TNF	Tumor Necrosis Factor
