# Supplementary material for: Skin Substitute Preparation Method Induces Immunomodulatory Changes in Co-Incubated Cells through Collagen Modification
Source: Pharmaceutics. 2021 Dec 15;13(12):2164. doi: 10.3390/pharmaceutics13122164 (PMC8705760; doi:10.3390/pharmaceutics13122164)
Supplement: Supplementary file 1 [file pharmaceutics-13-02164-s001.zip › pharmaceutics-1456207-supplementary.pdf]

pharmaceutics

MDPI

Supplementary Materials

Skin Substitute Preparation Method Induces Immunomodulatory Changes in Co-Incubated Cells through Collagen Modification

Jordan Holl [1], Cezary Pawlukianiec [1], Javier Corton Ruiz [1], Dawid Groth [1], Kamil Grubczak [1], Hady Razak Hady [2], Jacek Dadan [2], Joanna Reszec [3], Slawomir Czaban [4], Cezary Kowalewski [5], Marcin Moniuszko [1,6,*,†] and Andrzej Eljaszewicz [1,*,†]

[1] Department of Regenerative Medicine and Immune Regulation, Faculty of Medicine, Medical University of Bialystok, 15-269 Białystok, Poland; jordan.holl@umb.edu.pl (J.H.); cezary.pawlukianiec@gmail.com (C.P.); javiercortonruiz@gmail.com (J.C.R.); dawid.groth@umb.edu.pl (D.G.); kamil.grubczak@umb.edu.pl (K.G.)
[2] 1st Clinical Department of General and Endocrine Surgery, Faculty of Medicine, Medical University of Białystok, 15-276 Białystok, Poland; hadyrazakh@wp.pl (H.R.H.); jacek.dadan@umb.edu.pl (J.D.)
[3] Department of Medical Pathomorphology, Faculty of Medicine, Medical University of Białystok, 15-269 Białystok, Poland; joasia@umb.edu.pl
[4] Department of Anesthesiology & Intensive Therapy, Faculty of Medicine, Medical University of Białystok, 15-276 Białystok, Poland; slawomir.czaban@umb.edu.pl
[5] Department of Dermatology and Immunodermatology, Faculty of Medicine, Medical University of Warsaw, 02-091 Warsaw, Poland; ckowalewski@wum.edu.pl
[6] Department of Allergology and Internal Medicine, Faculty of Health Sciences, Medical University of Bialystok, 15-276 Bialystok, Poland
* Correspondence: Marcin.Moniuszko@umb.edu.pl (M.M.); Andrzej.Eljaszewicz@umb.edu.pl (A.E.); Tel.: +48-85-748-59-72 (M.M. & A.E.); Fax: +48-85-748-59-71 (M.M. & A.E.)
† Both authors contributed equally as senior authors.

Citation: Holl, J.; Pawlukianiec, C.; Corton Ruiz, J.; Groth, D.; Grubczak, K.; Hady, H.R.; Dadan, J.; Reszec, J.; Czaban, S.; Kowalewski, C.; et al. Skin Substitute Preparation Method Induces Immunomodulatory Changes in Co-Incubated Cells Through Collagen Modification. *Pharmaceutics* **2021**, *13*, 2164. https://doi.org/10.3390/pharmaceutics13122164

Academic Editors: Rossella Laurano and Monica Boffito

Received: 29 October 2021
Accepted: 9 December 2021
Published: 15 December 2021

Publisher's Note: MDPI stays neutral with regard to jurisdictional claims in published maps and institutional affiliations.

Copyright: © 2021 by the authors. Licensee MDPI, Basel, Switzerland. This article is an open access article distributed under the terms and conditions of the Creative Commons Attribution (CC BY) license (http://creativecommons.org/licenses/by/4.0/).

Abbreviations

CFSE–Carboxyfluorescein succinimidyl ester
ECM–Extracellular Matrix
hADM–Human-derived Acellular Dermal Matrix
IFNγ–Interferon Gamma
IL–Interleukin
LPS–Lipopolysaccharide
MFI–Mean Fluorescence Intensity
PBMC–Peripheral Blood Mononuclear Cells
TGF-β–Transforming Growth Factor Beta
Th1/17–T helper Types 1 or 17, respectively
TNF–Tumor Necrosis Factor

Table S1. hADM Decellularization Protocols.

hADM Preparation Method	Phase 1				Phase 2					Washing			Storage
	Reagents	time [h]	Temp. [°C]	[RPM]	Epidermal Removal	Reagents	time [h]	Temp. [°C]	[RPM]	Washing	Time [h]	[RPM]	Lyophilization Temp
hADM 1	1M NaCl + Antibiotic mix				Mechanical	SDS 0.1% + Antibiotic Mix							
hADM 2	1M NaCl + Antibiotic mix	24	37	40		3% Triton X-100 in PBS + Antibiotic Mix	24	37	40	H_2O	5 × 24h	60	YES/ −70
hADM 3	TrypLE Select + Antibiotic mix				Unnecessary	3% Triton X-100 in PBS + Antibiotic Mix							

NaCl—sodium chloride; SDS—Sodium dodecyl sulfate; PBS—Phosphate-Buffered Saline, H_2O—distilled deionized water.

Table S2. Antibodies Utilized in Flow Cytometry.

	Cellular Marker	Fluorochrome	Origin/Isotype	Clone	Supplier
T Cells	CD3	PerCP	Mouse / IgG1, k	SK7	BD
	CD4	FITC	Mouse / IgG2b, k	OKT4	BioLegend
	CD8	PE	Mouse / IgG1, k	SK1	BD
	CD25	PE-Cy7	Mouse / IgG1, k	BC96	BioLegend
	CD127	PE	Mouse / IgG1	R34.34	Beckman
	CD161	APC	Mouse / IgG1, k	HP-3G10	BioLegend
	CD196	PerCP Cy5.5	Mouse / IgG2b, k	G034E3	BioLegend
	IL-17	PE	Mouse / IgG1, k	N49-653	BD
	IFNγ	PE-Cy7	Mouse / IgG1, k	B27	BD
Monocytes	CD14	PerCP	Mouse / IgG2b, k	MϕP9	BD
	CD16	FITC	Mouse / IgG1, k	3G8	BD
	CD163	PE	Mouse / IgG1, k	GHI/61	BioLegend
	TIE-2	Alexa Fluor 647	Mouse / IgG1, k	Ab33	BioLegend
	IL-10	PE	Rat / IgG2a, k	JES3-19F1	BioLegend
	TNF	PE	Mouse / IgG1	6401.1111	BD

Table S3. Antibodies Utilized in Confocal Microscopy.

Primary Antibodies					
Marker		Origin/Isotype	Clone	Supplier	RRID
Collagen 1A1		Rabbit / IgG	Polyclonal	Invitrogen	AB_2547045
Collagen III		Rabbit / IgG	Polyclonal	Invitrogen	AB_2552139
Collagen IV		Mouse / IgG1	COL-94	Invitrogen	AB_558482
**Secondary Antibodies**					
**Fluorochrome**	**Reactivity**	**Origin/Isotype**	**Clone**	**Supplier**	**RRID**
Alexa Fluor 647	Mouse	Goat / IgG	Polyclonal	Invitrogen	AB_2536165
Alexa Fluor 700	Rabbit	Goat / IgG	Polyclonal	Invitrogen	AB_2535709

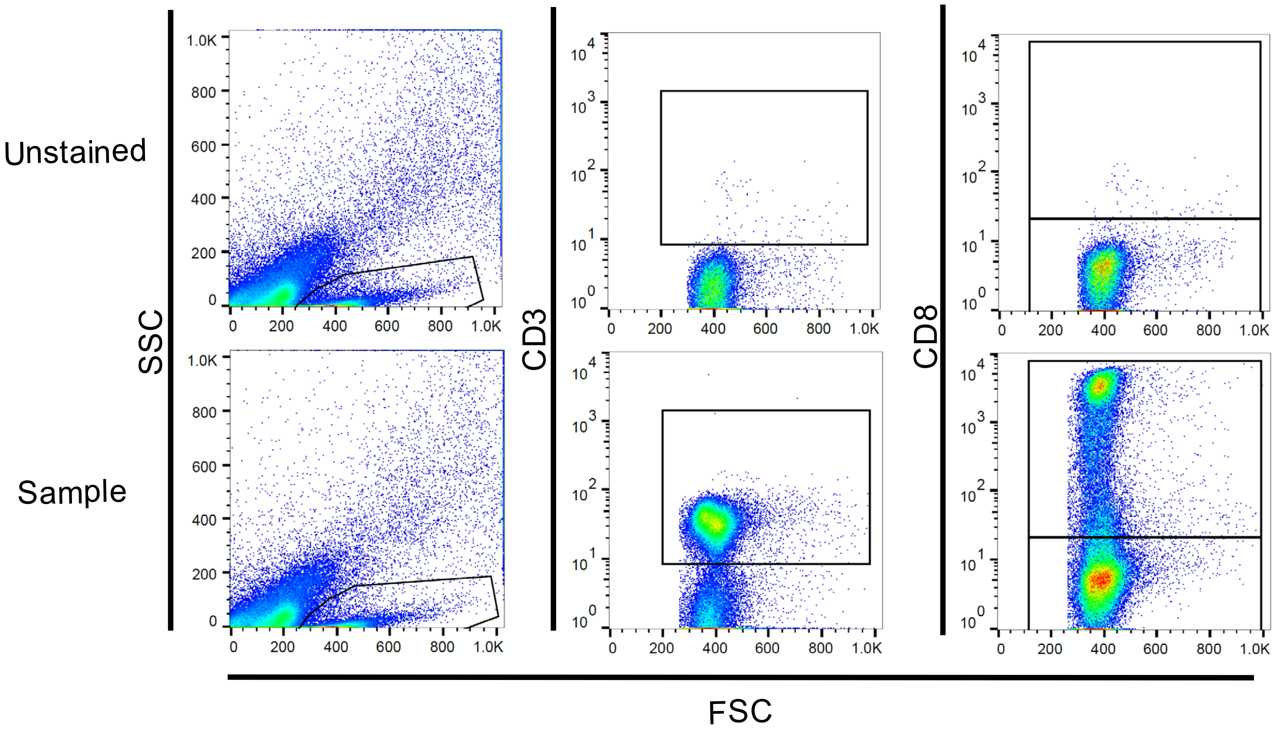

Figure S1. Representative Gating for T Cell Proliferation. This supplemental figure relates to Figure 1. CFSE-stained PBMCs cultured either alone or co-incubated with hADMs were collected, stained for CD3 & CD8, and underwent flow cytometry. Proliferation was quantified as shown on Figure 1 via histogram gating.

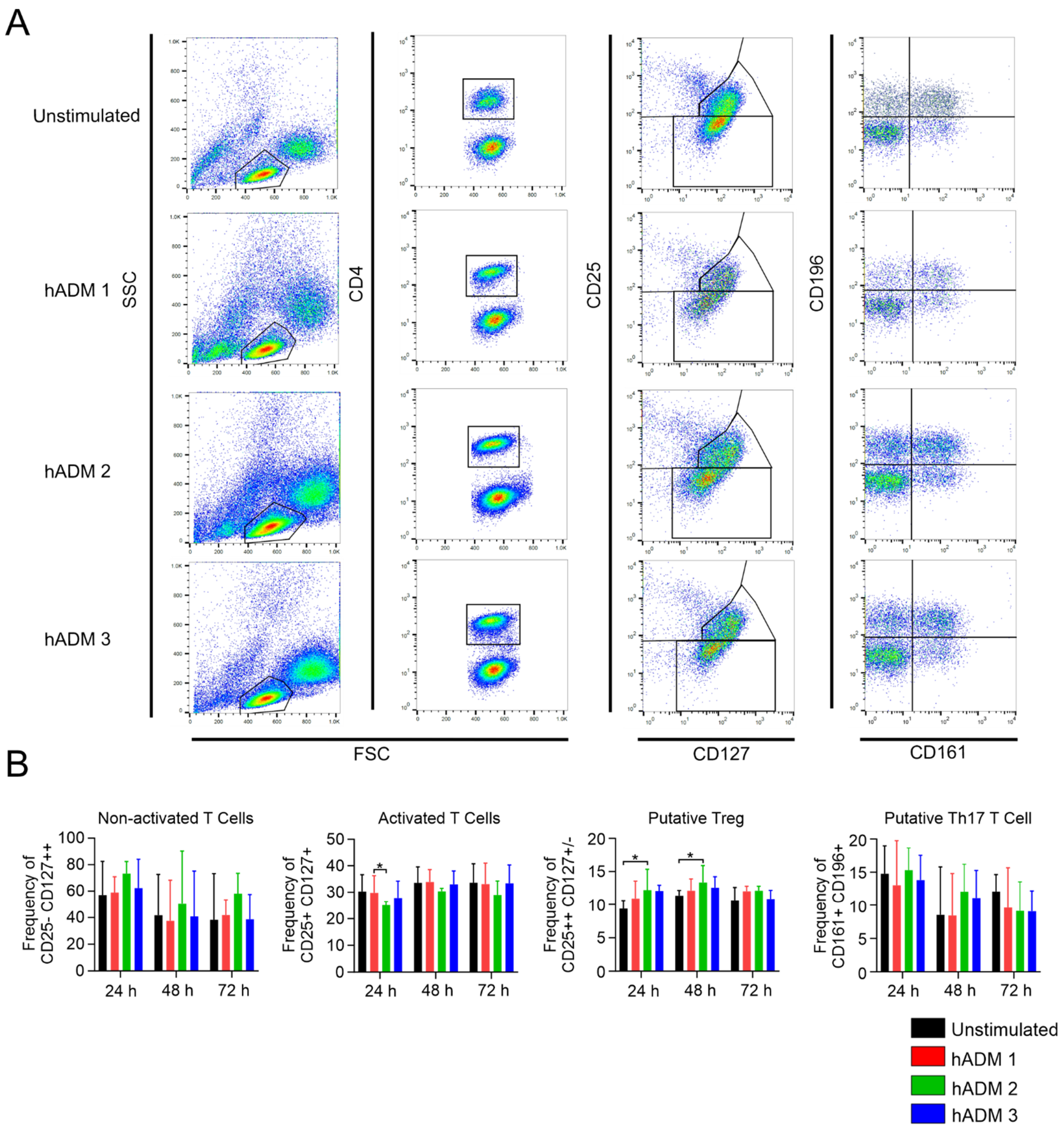

Figure S2. T Cell Phenotype is not Regulated by hADM Co-incubation. Healthy donor PBMCs were co-incubated alone or with hADMs for 1, 2 or 3 days. Next, cells were stained extracellularly and examined by flow cytometry. (**A**) Representative schematic of flow cytometry gating of extracellularly-stained T cell subsets. (**B**) Frequencies of T cell subsets were noted for inactive T cells (CD25−/CD127++), active T cells (CD25+/CD127+), and Tregs (CD25++/CD127+/−). Frequency of Th17 cells were also examined by CD196+/CD161+. Results expressed as medians + interquartile ranges. Two-tailed Wilcoxon matched-pairs signed rank test used for B. $n = 5$ with 2 technical replicates; * $p < 0.05$.

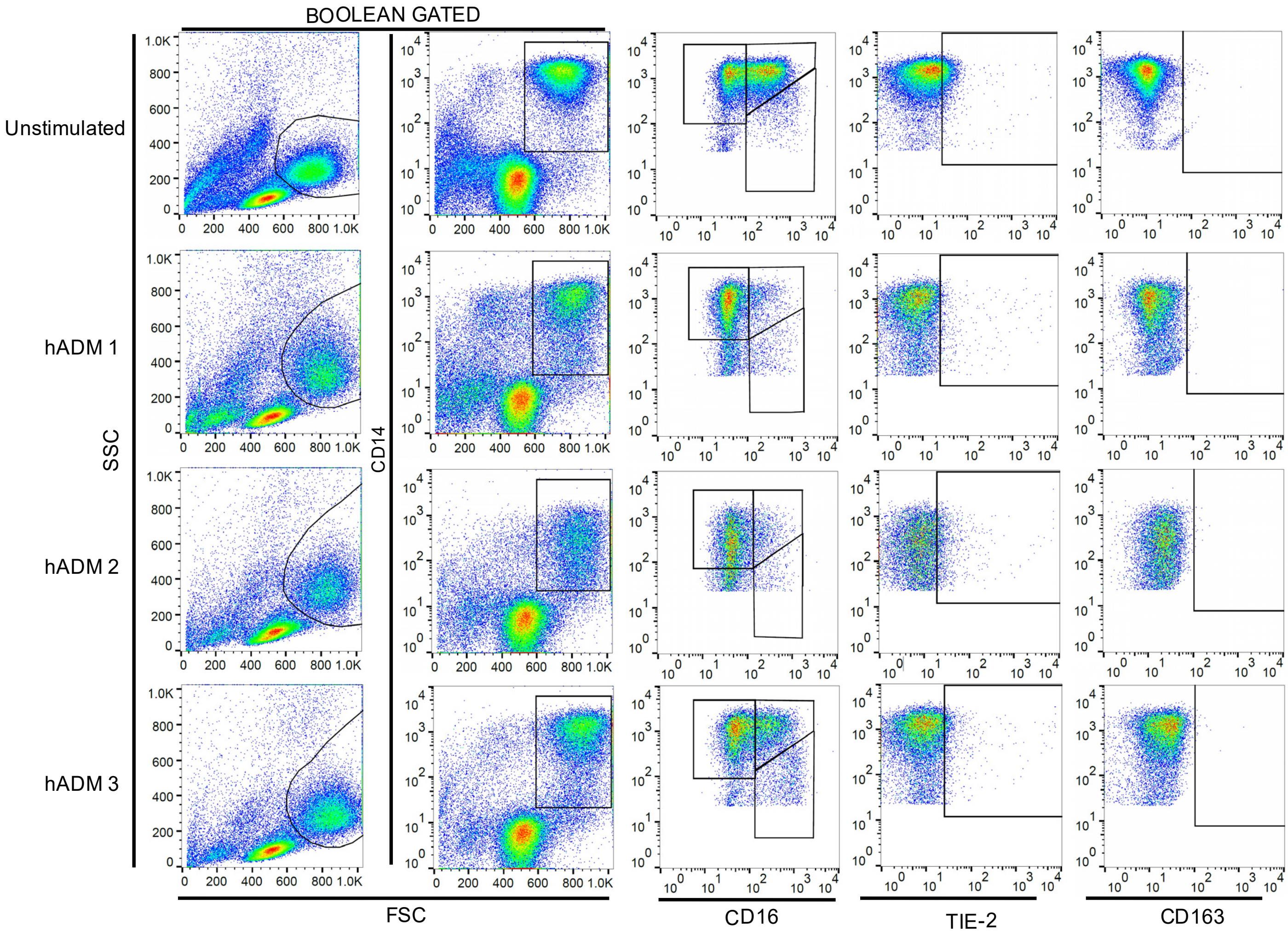

Figure S3. Representative Gating for Extracellularly-stained Monocytes. This supplemental figure relates to Figure 2. PBMCs cultured either alone or co-incubated with hADMs were collected, stained extracellularly for CD14, CD16, TIE-2 and CD163. Frequency of monocyte subsets (by CD14/CD16) as well as frequency and MFI of surface receptors TIE-2 & CD163 can be seen in Figure 2.

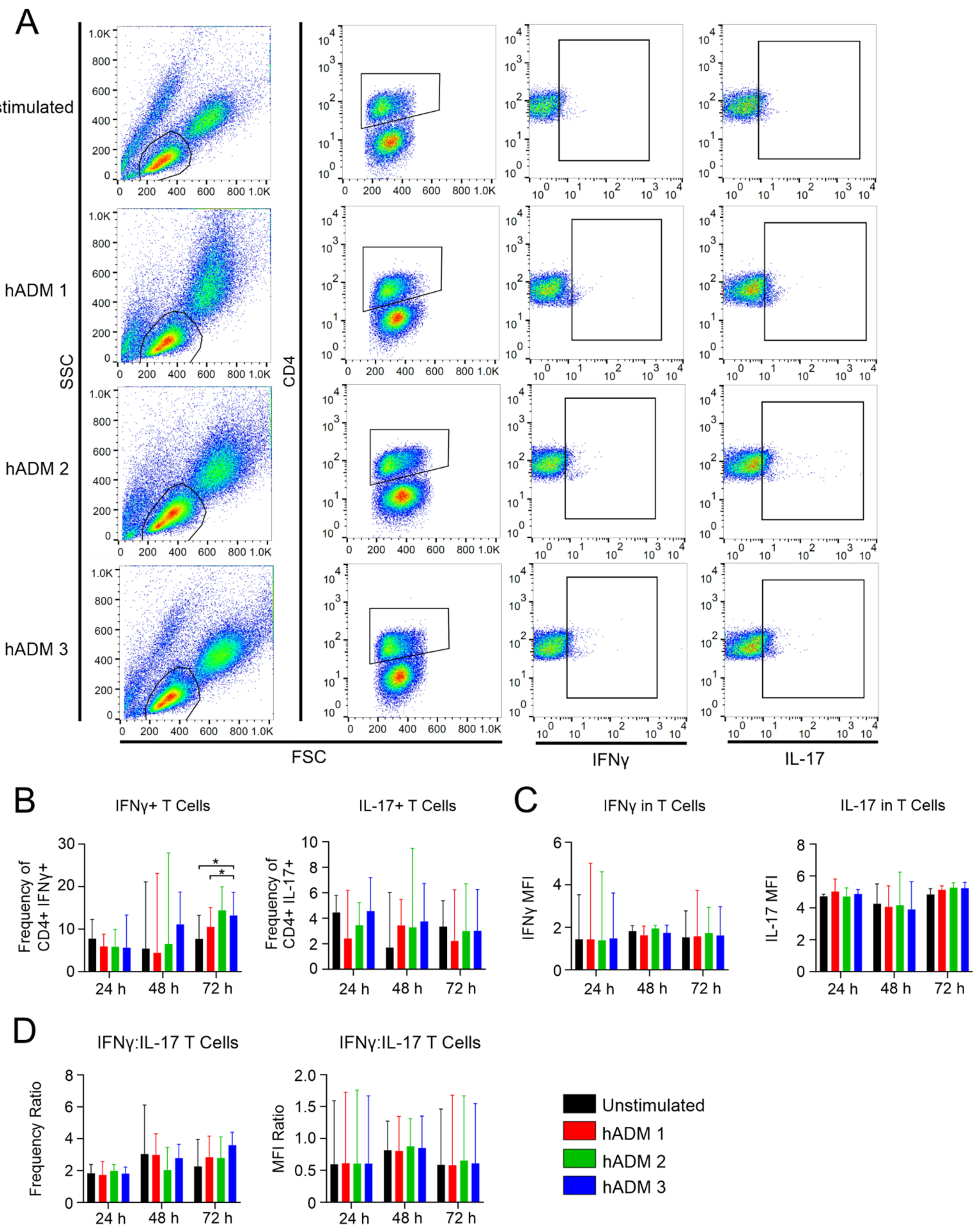

Figure S4. hADMs do not Induce Differential T Cell Function. Healthy donor PBMCs were co-incubated alone or with hADMs for 1, 2 or 3 days. 2 hours before collection, protein transportation was inhibited. Finally, cells were permeabilized and stained extracellularly and intracellularly before being examined by flow cytometry. (**A**) Representative schematic for flow cytometry gating. (**B**) Quantification of CD4+/IFNy+ or IL-17+ T cell frequency. (**C**) MFI quantification of CD4+/IFNy+ or IL-17+ T cells. (**D**) Frequency and MFI ratios of inflammatory T cell cytokines. Results expressed as medians + inter-quartile range. Two-tailed Wilcoxon matched-pairs signed rank test used for B–E. $n = 5$ with 2 technical replicates; $*p < 0.05$.

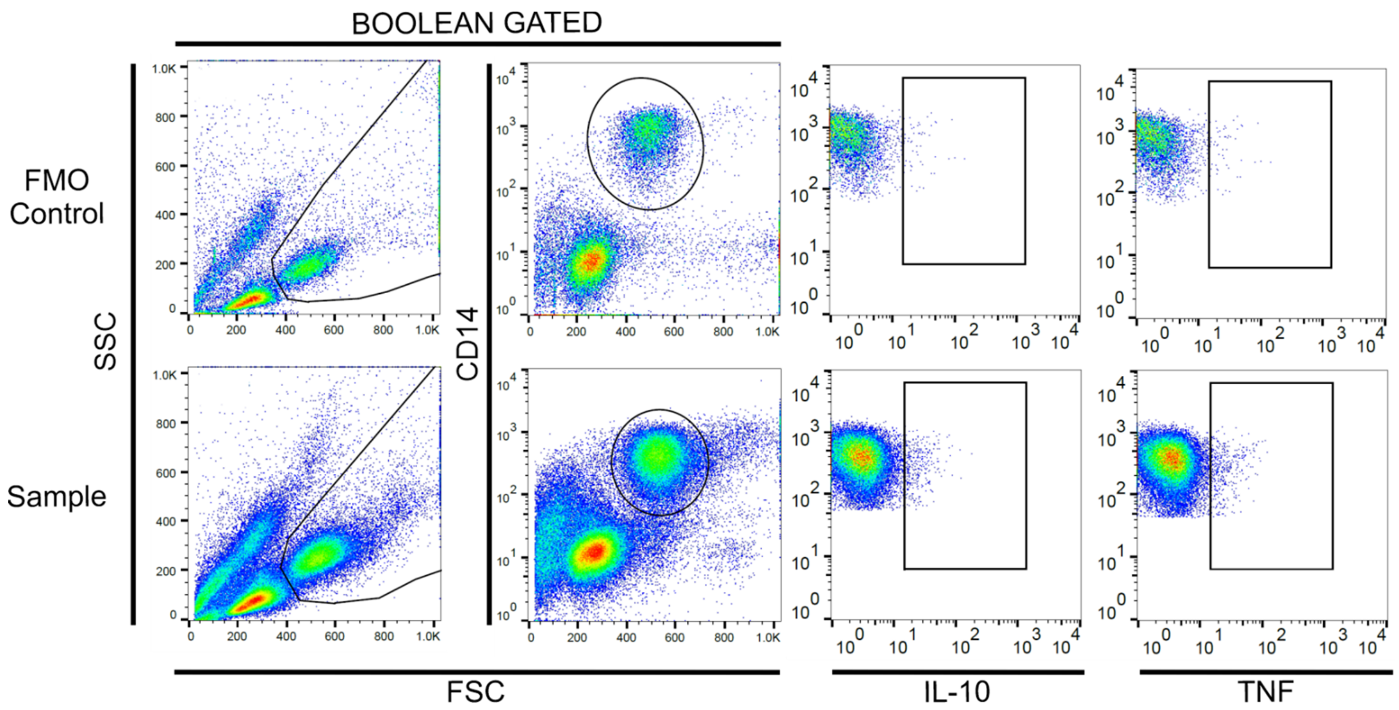

Figure S5. Representative Gating Strategy for Intracellularly-stained Monocytes. This supplemental figure relates to Figure 3. PBMCs cultured either alone or co-incubated with hADMs were collected, stained extracellularly for CD14, and intracellularly for IL-10 & TNF before undergoing flow cytometry. Quantifications of functional cytokines can be seen in Figure 3.

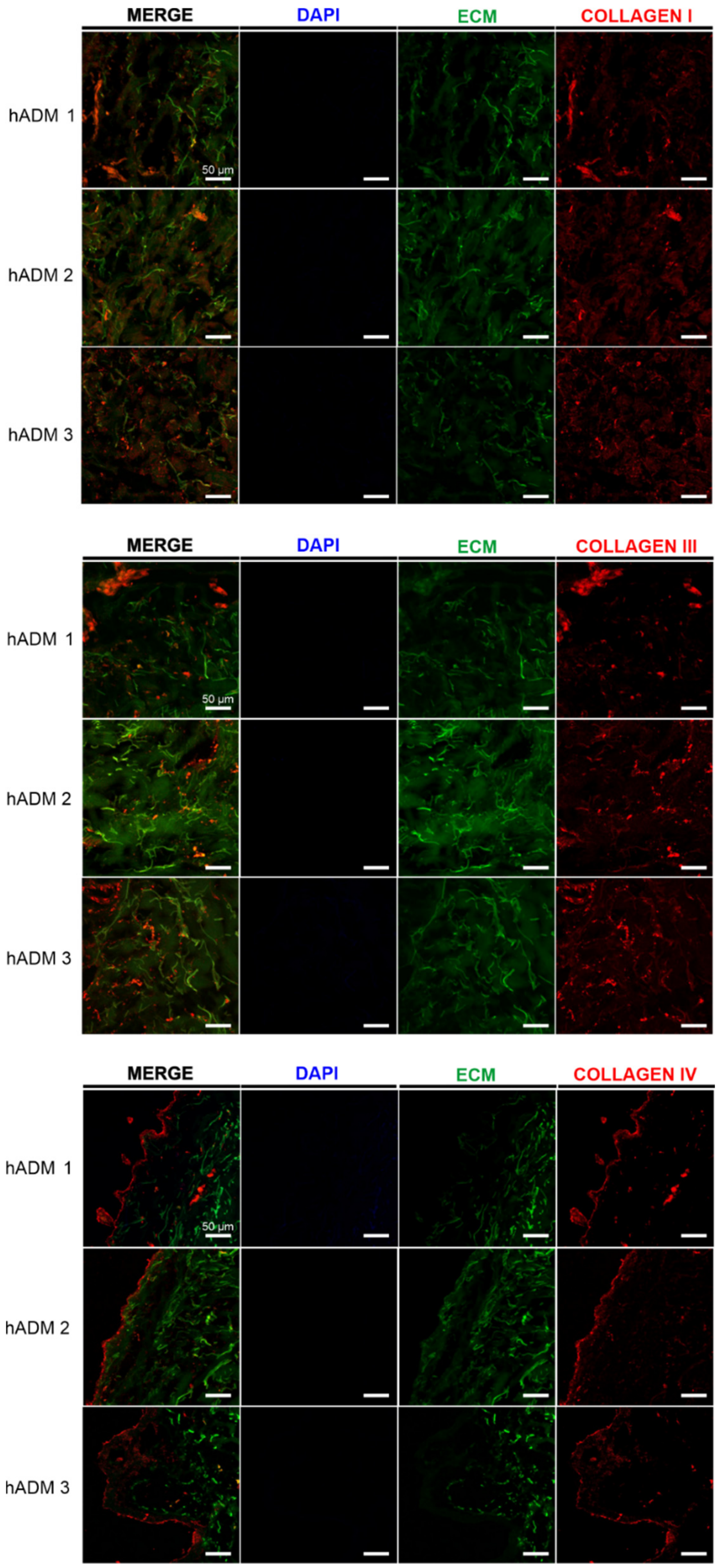

Figure S6. Representative Photos of ADMs without PBMC co-incubation. hADMs were examined confocally after fluorescence-conjugated antibody staining for collagens I, III and IV. Images for collagens I, III and IV can be seen for each hADM and in 3 separate channels as well as merged together. MERGE: Combined representation of channels 1–3, detailed below. Channel 1 (DAPI): cell nuclei staining via DAPI using a 405 nm laser. Channel 2 (ECM): Autofluorescent collagen fibers and ECM using a 488 nm laser. Channel 3 (Specific Collagen): Specific collagen fibers were stained using a primary and subsequently a secondary fluorescence-conjugated antibody using a 647 nm laser. All scale bars represent 50 µm.

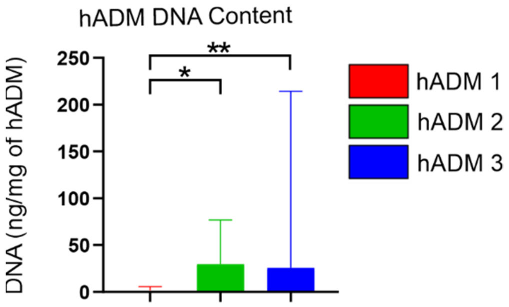

Figure S7. Quantification of residual DNA. hADMs were examined for their residual DNA content after processing. hADMs were entirely digested by proteinase K followed by DNA isolation and quantification. Results expressed as medians +/− interquartile ranges. One-tailed U Mann-Whitney test was used. $n = 4$ with 2 technical replicates; * $p < 0.05$, ** $p < 0.01$.
